# Fecal Calprotectin in Healthy Children Aged 1-4 Years

**DOI:** 10.1371/journal.pone.0150725

**Published:** 2016-03-07

**Authors:** Qingling Zhu, Feng Li, Junli Wang, Lixiao Shen, Xiaoyang Sheng

**Affiliations:** Department of Child and Adolescent Healthcare, MOE-Shanghai Key Laboratory of Children’s Environmental Health, Xinhua Hospital Affiliated to Shanghai Jiao Tong University School of Medicine, Shanghai, 200092, China; Nathan Kline Institute and New York University School of Medicine, UNITED STATES

## Abstract

**Objective:**

Calprotectin has been well emulated recently in adults as well as in children. The aim of this study was to assess fecal calprotectin concentrations in healthy children aged from 1 to 4 years.

**Methods:**

Volunteers were enlisted from 3 nurseries. A brief questionnaire was used to ensure these children meet the inclusion criteria, and some clinical and sociodemographic factors were collected. Anthro software (version 3.1) was used to calculated Length-for-age Z-scores (LAZ), weight-for-age Z-scores (WAZ), and weight-for-length Z-scores (WLZ) respectively. Fecal calprotectin was detected by a commercially available ELISA.

**Results:**

In total 274 children were recruited, with age ranging from 1 to 4 years old. The median FC concentration was 83.19 μg/g [range 4.58 to 702.50 μg/g, interquartile range (IQR) 14.69–419.45 μg/g] or 1.92 log10 μg/g (range 0.66 log10 to 2.85 log10 μg/g, IQR 1.17 log10-2.62 log10 μg/g). All of the children were divided into three groups, 1–2 years (12–24 months), 2–3 years (24–36 months), 3–4 years (36–48 months), with median FC concentrations 96.14 μg/g (1.98 log10 μg/g), 81.48 μg/g (1.91 log10 μg/g), 65.36 μg/g (1.82 log10 μg/g), respectively. There was similar FC level between boys and girls. FC concentrations showed a downward trend by the growing age groups. A statistic difference was found in FC concentrations among groups 1–2 years, 2–3 years and 3–4 years (*P* = 0.016). In inter-groups comparison, a significant difference was found between children aged 1–2 years and children aged 3–4 years (*P* = 0.007). A negative correlation trend was found between age and FC concentration (Spearman's rho = -0.167, *P* = 0.005) in all the participants. A simple correlation was performed among WLZ, WAZ, birth weight, or birth length with FC, and there was no correlation being observed.

**Conclusion:**

Children aged from 1 to 4 years old have lower FC concentrations compared with healthy infants (<1years), and higher FC concentrations when comparing with children older than 4 years and adults.

## Introduction

Calprotectin, also known as S100A8/A9, is a small, calcium- and zinc-binding protein with a molecular weight of 36.5 KDa [1, 2]. It belongs to the S100 family, which constitutes approximately 60% of the soluble cytosol proteins in human neutrophil granulocytes [3]. Twenty-five tissue- and cell-specific S100 proteins have been recognized to date in humans, and the vast majority of these are complex in form [4]. Although the exact biological function of calprotectin is still unclear, it has been reported to have bactericidal and fungicidal properties [5]. Since 1992, when a fecal calprotectin (FC) ELISA became available [6], the number of studies of FC concentrations in infectious diseases has increased, especially in cases of intestinal infectious diseases [7]. Calprotectin may partly explain the high Ca^2+^ concentration in the gut lumen because it makes calprotectin resist proteolytic degradation [8, 9] and calprotectin is a very stable component when bound to calcium. It can remain in the stool for one week at room temperature without any significant degradation [6, 10] and can be measured using a simple ELISA test within hours, resulting in a quick turnaround of results that makes it efficient for clinical decision making. The endoscope remains the gold standard method for assessing intestinal inflammation; however, because endoscopic examinations are invasive, expensive and uncomfortable, a non-invasive, inexpensive, simple and sensitive maker for detecting and monitoring the occurrence and development of intestinal inflammation diseases is greatly needed. Within this context, a large number of studies have focused on the use of FC concentrations to quantify intestinal inflammation. FC concentration has been associated with the degree of disease activity in inflammatory bowel disease in both adults [11–13] and children [14–16], and it has been regarded as a good marker for evaluating intestinal inflammation [17, 18].

Because calprotectin research has become increasingly common, it is meaningful to determine reference values for calcium protein. Among the vast majority of studies, the FC levels for adults and children, and for children of different ages, differ dramatically. Studies of FC concentrations in adults, children older than 4 years, and healthy infants are somewhat common; however, studies of children aged younger than 4 years are still unclear and scarce. In continuation of our previous study [19], this study aimed to establish a baseline for FC concentration in healthy children aged 1 to 4 years old to describe the FC concentrations of children in that age range.

## Participants and Methods

### Volunteer recruitment

The eligible children were recruited from 3 nurseries between November 2014 and February 2015 and received a conventional check-up at the Department of Children and Adolescent's Health Care at Xinhua Hospital. During the children’s check-up time, the child’s primary guardian was asked to complete a brief questionnaire regarding several clinical and sociodemographic factors, including gestational age, birth weight, sex, 5-minute Apgar score, postnatal age, neonatal diseases, and the weight and length of the child at the time of specimen collection. The inclusion criteria were as follows: 1) age range 1 to 4 years (12–48 months); 2) born at a gestational age >37 weeks; 3) 5-minute Apgar score >7; 4) birth weight range 2,500 g to 4,000 g; 5) no known underlying chronic inflammatory disease or any signs or symptoms of infection (cold, flu or eczema) or gastrointestinal disease (diarrhea, vomiting, hematochezia or fever); and 6) no use of drugs, such as steroidal or non-steroidal anti-inflammatory drugs, gastric acidity inhibitors or antibiotics, in the past two weeks. This study was conducted at the Department of Child and Adolescent Health Care of Xinhua Hospital, Shanghai, Jiaotong University School of Medicine.

A total of 280 children were invited to our study; 6 (2.14%) potential subjects were excluded by reason of premature birth (1 case), suffering from eczema (3 cases) and vomiting (2 cases). Ultimately, 274 apparently healthy children were recruited for our study and submitted fecal samples. The children were divided into three age groups: 1–2 years (12–24 months), 2–3 years (24–36 months) and 3–4 years (36–48 months).

### Sample collection

The guardian of each child was offered a plastic screw-capped container and given thorough instructions about stool sample collection. The samples were brought directly to us or were delivered by mail that day or the next day. The fecal samples were stored at– 80°C.

### Fecal calprotectin measurement

The stool samples were thawed at room temperature before examination. A commercially available ELISA test was used to quantitatively measure the concentrations of fecal calprotectin (Bühlmann Laboratories AG, Schönenbuch, Switzerland), as described previously [20–22], using a simple extraction procedure. Each sample runs with blanks, standards and controls. The FC levels are expressed as μg/g per sample. If a sample yielded a reading higher than the maximum calibration level (600 μg/g), the remaining extract was further diluted 1:6 with incubation buffer, and a repeated detection was performed.

### Somatometry and calculation

All of the recruited children were measured using a standard anthropometric scale. Because of the children’s age differences, the measurement methods differed somewhat. In children younger than 2 years, a recumbent position measurement method was adapted to measure supine lengths (Seca Corp, Hanover, MD, USA, precision 0.1 cm) and weights (precision 5 g). Children older than 2 years were measured using a vertical children's electronic scale to record their heights (Seca Corp, Hanover, MD, USA, precision 0.1 cm) and weights (precision 5 g). All weight and height measurement was performed by two well-trained members of our research team. Anthro software (version 3.1) was used to calculate length-for-age Z-scores (LAZ), weight-for-age Z-scores (WAZ), and weight-for-length Z-scores (WLZ) in accordance with the World Health Organization Child Growth Standards.

### Ethical considerations

We obtain informed consent from the guardians on behalf of the children enrolled in your study. The consent on behalf of the children enrolled was written. This study was approved by the Institutional Ethics Committee of Xinhua Hospital (approval No. XHEC-D-2014-073, and carried out in accordance with the revised Declaration of Helsinki.

### Statistical Analysis

The statistical analysis was performed using the SPSS version 16.0 software package for Windows (SPSS, Inc., Chicago, IL, USA). Median values were used because a skewed distribution might exist in the FC concentrations. The FC concentrations were also transformed to their base 10 logarithms (log10). The FC values of the different age groups were compared using the Mann-Whitney U test and Kruskal-Wallis H test. All of the tests were 2-sided, and differences were considered significant when *P*-values were 0.05 or less. A simple regression analysis was carried out to estimate the correlations of WLZ, WAZ, birth weight, or birth length with the FC concentration. Spearman’s correlation test was used to determine the relationship between age and FC values.

## Results

The study recruited a total of 274 children aged from 1 year to 4 years. The genders were unequally represented in the study, with 168 boys (61.31%) and 106 girls (38.69%). The baseline characteristics of all of the volunteers, including age, gestational age, birth weight, birth length, LZA, WAZ and WLZ are shown in Table 1 (S1 Essential Data).

**Table 1:** Participant information.

Group, years	Age	Gestational age	Birth weight	Birth length	LAZ*	WAZ*	WLZ*
	Median (range)	Median (range)	Median (range)	Median (range)	Mean±SD	Mean±SD	Mean±SD
1–2	18.20(12.02–24.00)	39.00(37.00–42.00)	3310.00(2500.00–4450.00)	50.0(46.0–55.0)	0.84±0.88	0.51±1.02	0.43±0.80
2–3	31.25(24.10–35.83)	39.00(37.00–43.70)	3350.00(2530.0–5500.00)	50.00(47.00–54.00)	0.33±0.98	0.30±0.94	0.02±1.01
3–4	40.70(36.13–48.17)	38.40(37.00–40.90)	3500.00(2500.00–4500.00)	50.00(46.00–54.00)	0.40±1.11	0.47±0.89	0.55±0.96
Total	29.40(12.02–48.17)	39.00(37.00–43.70)	3352.00(2500.00–5500.00)	50.00(46.00–55.00)	0.56±1.00	0.42±0.97	0.37±0.92

*LAZ, WAZ and WLZ were calculated using the height (length) and weight measured when the fecal samples were collected.

**Fig 1:**
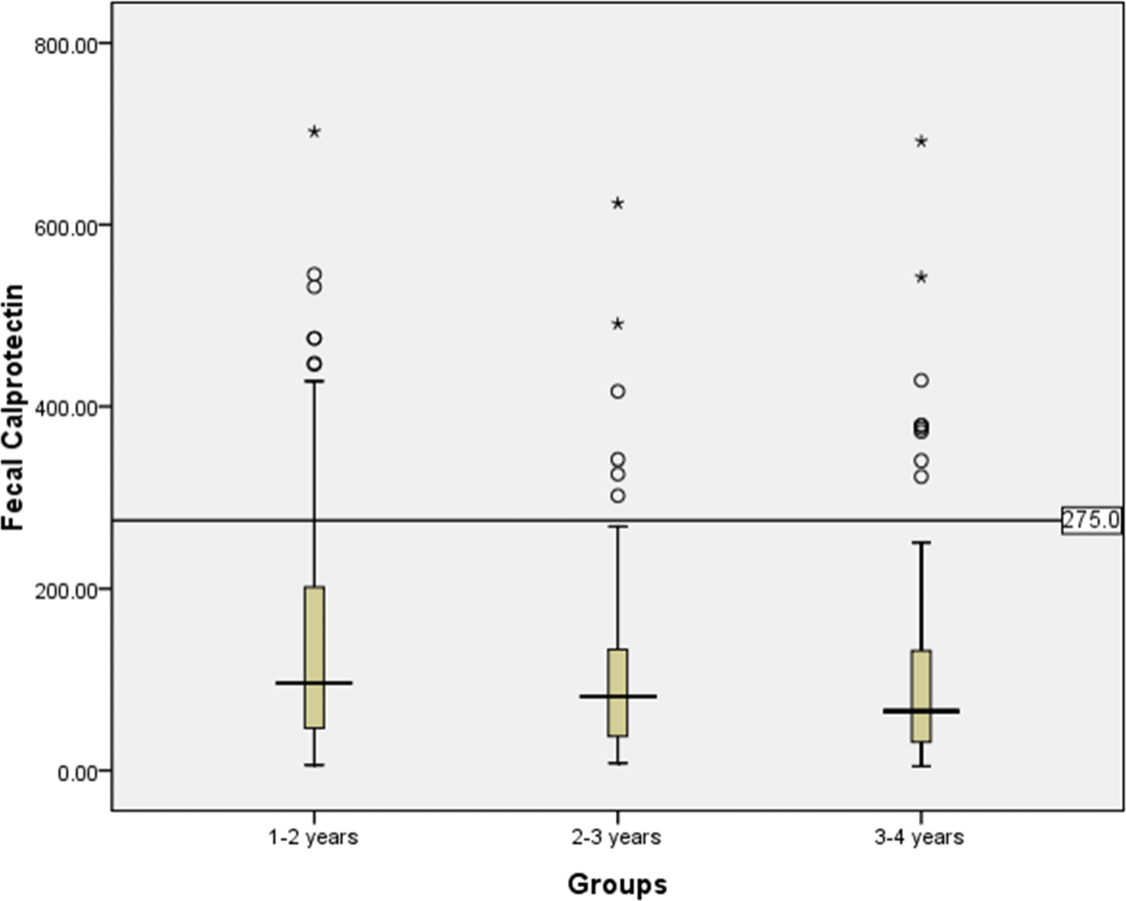
Fecal calprotectin concentrations in three age groups of healthy children.

The median FC concentrations of all of the participants were 83.19 μg/g (range 4.58 to 702.50 μg/g, interquartile range [IQR] 14.69–419.45 μg/g, 95% percentile 419.45 μg/g, mean±1.96 standard deviation [SD] 129.26±255.21 μg/g) or 1.92 log10 μg/g (range 0.66 log10 to 2.85 log10 μg/g, IQR 1.17 log10-2.62 log10 μg/g). The FC concentrations were unevenly distributed in the three subgroups, with median values of 96.14 μg/g (95% percentile 447.73 μg/g, mean±1.96SD 150.84±270.26 μg/g) for the 1–2 years group, 81.48 μg/g (95% percentile 368.04 μg/g and mean±1.96SD 113.42±223.75 μg/g) for the 2–3 years group, and 65.36 μg/g (95% percentile 379.33 μg/g and mean±1.96SD 112.05±252.60 μg/g for the 3–4 years group, respectively. The FC concentrations showed a downward trend with increasing age. These results are presented graphically in Table 2 and Fig 1 (S1 Essential Data).

**Table 2:** Fecal calprotectin concentrations in apparently healthy children.

Age, years	Boys		Girls		All		
	N	Median FC(5th-95th), μg/g	N	Median FC(5th-95th), μg/g	N (%)	Median FC(5th-95th), μg/g	Median FC(5th-95th), log10 μg/g
1–2	72	93.36(19.11–446.68)	47	122.79(12.27–475.02)	119(43.43)	96.14(19.67–447.73)*	1.98(1.29–2.6)
2–3	50	71.48(14.38–333.19)	22	93.77(24.49–479.87)	72(26.28)	81.48(16.61–368.04)	1.91(1.22–2.5)
3–4	46	63.82(8.45–485.32)	37	65.37(13.37–349.33)	83(30.29)	65.36(9.78–379.33)*	1.82(0.99–2.5)
*H* value		4.547		5.591		8.250	8.250
*P*		0.103		0.061		0.016	0.016

*The FC level of the children aged 1–2 years was significantly different from that of the children aged 3–4 years, *P* = 0.007. 5th-95th: 5th to 95th percentile, FC: fecal calprotectin

In each subgroup, no statistically significant difference in FC concentration between genders was found (*P* all >0.05). In all subjects, there was no difference in the FC concentration between the boys and the girls; the boys' median FC was 76.19 μg/g (IQR 13.03–415.14 μg/g), and the girls' was 89.75 μg/g (IQR 14.95–424.49 μg/g; *P*>0.05). There was a statistically significant difference in the FC concentrations among the three age groups (*P* = 0.016). In an inter-groups comparison, a significant difference was found between the children aged 1–2 years and the children aged 3–4 years (*P* = 0.007), while no statistically significant difference in FC concentrations was found between the children aged 1–2 years vs. 2–3 years or between the children aged 2–3 years vs. 3–4 years. The median FC concentrations of all of the groups are shown in Table 2. A negative correlation trend was found between age and FC concentrations (Spearman's rho = -0.167, *P* = 0.005) for all of the participants.

There was no correlation found when a simple correlation of WLZ, WAZ, birth weight, or birth length with FC was analyzed (all *P*>0.05). Four percent of all the participants (13 cases, 4.74%) had an FC concentration >419 μg/g (95% reference range in children with 1–4 years old), including 8 cases in group 1–2 years, 2 cases in group 2–3 years and 3 cases in group 3–4 years. Twelve percent of all the participants (35 cases, 12.77%) had an FC concentration >275 μg/g, which has been declared as the upper limit for children older than 1 year [23]. These 35 cases received telephone follow-up, and none were found to be taking drugs or suffering from gastrointestinal disease or respiratory infections.

## Discussion

FC levels are already being widely used in the monitoring of inflammatory bowel disease, infectious colitis, irritable bowel syndrome and ulcerative colitis in adults [24, 25]. Because FC level detection is non-invasive and convenient, this highly significant research has been extended to children as well. A systematic review by Kostakis et al [26] proposed that FC testing could be used to support diagnoses or confirm a relapse of inflammatory bowel disease in children. FC is also becoming increasingly measured in other gut inflammatory conditions that affect children such as cystic fibrosis. In addition, FC was uniquely the only biomarker elevated compared to other inflammatory stool markers in cystic fibrosis (these others were not elevated) [27]. Some scholars have proposed that FC concentration is a good predictor of colorectal inflammation in children with gastrointestinal symptoms [16]. Additionally, FC level can be a direct measure of intestinal inflammation and can detect a risk of histological relapse in pediatric IBD patients. In 2008, Diamanti et al [28] published a retrospective thesis that included 73 children (1.5–18 years) with a first histological examination showing quiescent IBD and a second histological examination over the next 3 years. After a 36-month follow-up, the researchers found that a significantly higher number of relapsed children than non-relapsed children had FC concentrations >275 μg/g. In view of the close relationship between FC levels and diseases, and because the FC levels of children of different ages were still unclear, information about baseline FC concentrations in healthy children of different ages is urgently needed. In continuation of our previous study [19], the FC concentrations of children with aged 12 to 48 months (1–4 years) were analyzed in this study. To the best of our knowledge, this is the first time that the FC concentrations of the children in this age range in Shanghai, China have been reported.

Ezri et al [23] advised that FC concentrations should vary with age: for children during the first year of life, < 350 μg/g; for older children, < 275 μg/g; and for adults, < 50 μg/g. Compared with the infants younger than 1 year old in our previous study (which found median FC concentrations of 375.2 μg/g in children aged 1–3 months, 217.9 μg/g in children aged 3–6 months, 127.7 μg/g in children aged 6–9 months and 96.1 μg/g in children aged 9–12 months) [19], the FC level in children aged 1–4 years was lower. Moreover, different FC concentrations were found in the presumably healthy children within each age group in our study, with median FC concentrations of 96.14 μg/g in children aged 1–2 years, 81.48 μg/g in children aged 2–3 years and 65.36 μg/g in children aged 3–4 years. There was a significant difference between the 1–2 year group and the 3–4 year group, which was impressive. The FC concentrations were similar for the children aged 1–2 years versus those aged 2–3 years and for the children aged 2–3 years versus those aged 3–4 years. This result indicated that more than one reference ranges are needed for healthy children aged 1 to 4 years, consistent with the conclusions of Oord’s study [29] in 2014. However, Oord et al [29] reported that the median FC concentrations were 192 mg/kg (192 μg/g), 72 mg/kg (72 μg/g), 47 mg/kg (47 μg/g), 31 mg/kg (31 μg/g) and 36 mg/kg (36 μg/g) for children aged 1–6 months, 6–12 months, 1–2 years, 2–3 years and 3–4 years, respectively, which was generally lower than for the same-aged children in our study. Hestvik et al [30] demonstrated a median FC concentration of 75 mg/kg (75 μg/g), range 53 to 119 mg/kg (53–119 μg/g), in children aged 1 to 4 years old in a low-income country in sub-Saharan Africa. Compared to this study, the median of FC concentrations in the children aged 1–2 years and 2–3 years in our study was higher. Our previous research [21], which compared the FC concentrations of children aged 0–6 months in rural (Yunnan China) and urban (Shanghai China), found that children in high-poverty areas and those who were smaller than expected for their age had higher FC concentrations. This may be caused by chronic intestinal inflammation. However, children in Shanghai, China grow well, as being founded in comparison with the World Health Organization standards [31]. These children should not suffer from chronic intestinal inflammation. Sub-Saharan Africa may provide "a more favorable environment or a protective effect associated with increasing age" than Shanghai does, but the precise reason for the high FC concentrations among the children from Shanghai was unclear. The FC concentration of all of the participants was 83.19 μg/g, higher than that reported by Fagerberg et al [20], who claimed that the baseline value for adults (< 50 μg/g) could also be used for children aged 4 to 17 years. Considering past research, such as Ezri's [23] reports for infants, children and adults, Fagerberg's [20] reports for children aged 4–17 years and our previously research [19], we submit FC concentrations <419 μg/g (higher than 275 μg/g) as a baseline for children aged 1–4 years and suggest long-term follow-up to rule out intestinal inflammation in children within that age range whose FC levels are higher than 419 μg/g.

The spontaneous normalization of FC concentrations without disease has been reported [20]. In our study, all 35 of the participants whose FC concentrations were higher than 275 μg/g received a telephone follow-up. However, no abnormal symptoms, such as respiration infection, stomachache, diarrhea or abnormal crying, were reported by the guardians. We did ensure that children taking non-steroidal anti-inflammatory drugs were excluded from our study because these medications have been reported to elevate FC concentrations [32]. The high FC levels in patients with IBD were deemed to result from an increased turnover of leukocytes in the intestinal wall and the migration of neutrophils into the enteric cavity, a finding that was supported by Roseth et al [33] and Tibble et al [18] in their studies about the correlation between the excretion of indium-111-labeled neutrophils and FC concentrations. Considering this finding, we could not rule out the possibility that the children with high FC concentrations could develop intestinal inflammation in the near future; therefore, parents should pay close attention to this possibility. There were no gender differences in FC concentrations among healthy children, as previous studies have shown [19, 20, 29].

It has been reported that calprotectin shows 14 potential cleavages sites for trypsin in human. Trypsin activities are maximal in the proximal part of the intestinal tract and drop rapidly in the more distal segments [34]. In our present study, the stool samples we collected were all excreted in vitro, that is, they were subjected to trypsin when went through whole digestive tract. Thus the level of calprotectin in the stool samples may be lower than that directly draw from intestinal [35].

## Limitations

There were some limitations to our study. First, because this was a cross-sectional study, the FC levels of the participants were not dynamically monitored. Second, although the children with FC concentrations >275 μg/g received a follow-up phone calls, no related examinations, such as diagnostic colonoscopy or esophagogastroduodenoscopy, was performed to determine the causes of the elevated FC levels. A simple follow-up phone call is not enough to say these children are completely healthy. We may only comment that they do not have any serious symptomatic gastrointestinal disease at that time. Third, there is a limitation to convince for the small sample size, larger sample are needed to build a FC reference ranges in children aged 1–4 years. Last but not least, we only test apparently healthy children and no children with intestinal inflammation were recruited; the overlap of values between normal and abnormal was not explored, which need to be added in our following research.

## Conclusion

Our data confirm that children aged from 1 to 4 years have lower FC concentrations compared with healthy infants and higher FC concentrations compared with children older than 4 years and adults. We hope the range of reference values reported in our study can be used in further studies concerning FC concentrations and disease in children within this age range.

## Supporting Information

S1 Essential Data (SAV)
